# Missed and misdiagnosis of immunodeficiency: Good syndrome presenting as persistent COVID-19 shedding

**DOI:** 10.5415/apallergy.0000000000000171

**Published:** 2025-01-08

**Authors:** Valerie Chiang, Tiffany C. H. So, Philip H. Li

**Affiliations:** 1 Department of Pathology, Queen Mary Hospital, Hong Kong SAR, China; 2 Division of Rheumatology & Clinical Immunology, Department of Medicine, Queen Mary Hospital, The University of Hong Kong, Hong Kong SAR, China

**Keywords:** COVID-19, Good syndrome, Immunodeficiency, Persistent, Shedding

## Abstract

A 61-year-old man was referred to the immunology clinic due to prolonged COVID-19 infection and absent severe acute respiratory syndrome coronavirus 2 (SARS-CoV-2) antibodies. His infection, confirmed by polymerase chain reaction (PCR) testing upon returning to Hong Kong from Vancouver, resulted in a 31-day hospitalization due to persistent positive PCR results, despite his remaining asymptomatic. Laboratory tests revealed lymphopenia, low immunoglobulin levels, and unremarkable serum protein electrophoresis, with all viral serologies, including HIV, being negative. Although he received 3 doses of the Pfizer BioNTech vaccine, he showed no detectable SARS-CoV-2 antibodies. Further evaluation indicated almost-absent CD19 cells and low CD4 and CD8 counts, and coupled with his history of thymoma, this led to a diagnosis of Good syndrome. The patient began subcutaneous immunoglobulin replacement therapy and was started on long-term valganciclovir for moderate cytomegalovirus antigenemia.

## 1. Introduction

Good syndrome (GS) is a rare immunodeficiency disorder characterized by thymoma and hypogammaglobulinemia, leading to various clinical manifestations including infections and autoimmunity [1–3]. Initially considered as a subgroup of common variable immunodeficiency (CVID), GS is now classified as an acquired form of combined immunodeficiency affecting middle-aged to older adults [1, 4]. However, its underlying pathogenesis remains poorly understood [5]. With the lack of formal diagnostic criteria, its recognition is challenging and often delayed by years [3]. At the time of diagnosis, patients usually present with recurrent infections, hypogammaglobulinemia, and reduced B-cell counts, but other symptoms and clinical features may precede these findings [1]. We describe a case of GS referred for prolonged, asymptomatic COVID-19 infection.

## 2. Case report

A 61-year-old man was referred to the immunology clinic for prolonged COVID-19 infection and absent severe acute respiratory syndrome coronavirus 2 (SARS-CoV-2) antibodies. His initial infection was diagnosed by polymerase chain reaction (PCR) testing upon his return to Hong Kong from Vancouver and was admitted to the Hong Kong Infection Control Centre. He remained asymptomatic during his entire hospitalization, however, was unable to be discharged due to persistent positive throat swab PCR results (SARS-CoV-2 T478K and N501Y variants) until 31 days later, when he finally tested negative. Bloodwork revealed lymphopenia (0.5 × 10^9^/l, reference range [RR]: 1.06–3.61) with normal white cell count (4.02 × 10^12^/l, RR: 3.89–9.93) and neutrophil count (2.90 × 10^9^/l, RR: 2.01–-7.42), low immunoglobulin (Ig) A (52 mg/dl, RR: 70–386), IgG (662 mg/dl, RR: 819–1725), and IgM (61 mg/dl, RR: 55–307) levels. Serum protein electrophoresis was unremarkable. Other viral serologies and HIV were negative.

The patient received 2 doses of the Fosun-Pharma Pfizer BioNTech vaccine (Shanghai, China) 6 months prior and a third BioNTech booster dose 5 months after his infection. Despite these vaccinations and infection history, he had absent SARS-CoV-2 antibodies, including nucleocapsid protein IgG, RBD (receptor-binding domain) IgG, and RBD IgM. He was then referred to the specialist immunology clinic for assessment.

The patient had no significant medical history, apart from a thymoma diagnosed in 2013, for which he underwent thymectomy and adjuvant low-dose radiation therapy without any complications. He had no myasthenia gravis or other autoimmune phenomenon. He denied any other serious, prolonged, unusual, or recurrent infections until his episode of COVID-19. He had no history of malignancy nor treatment with immunosuppressive therapy. His parents were nonconsanguineous, and his family history was unremarkable.

Further, lymphocyte subset enumeration revealed almost-absent CD19, low CD4 and CD8 (Table 1). Lymphocyte proliferation also showed decreased proliferation response to phytohemagglutinin, concanavalin A, and pokeweed mitogen, suggesting defective response in both T and B lymphocytes (Table 2). A pneumococcal vaccine challenge with PPSV23 (23-valent pneumococcal polysaccharide vaccine) was performed, which showed no notable increase in protective antibody titers in any serotype, even after PCV13 (13-valent pneumococcal conjugate vaccine) vaccination. Combined with his history of thymoma and functional antibody deficiency, he was diagnosed with GS and started on subcutaneous Ig replacement therapy. Autoimmune screening (including antiacetylcholine receptor antibodies) was unremarkable. He was also noted to have moderate *cytomegalovirus* (CMV) pp65 antigenemia (7 Pos/2 × 10^5^ white blood cells) and was started on long-term valganciclovir.

**Table 1.** Lymphocyte subset of the patient

		Reference range
B-cells (CD19) %	0.1 L%	6.6–22.6
B-cells (CD19) no.	0 L/µl	160–708
T-cells (CD3) %	83.7 H%	47.2–79.4
T-cells (CD3) no.	542 L/µl	716–2011
Th (CD4) %	28.5%	23.1–46.1
Th (CD4) no.	181 L/µl	415–1418
Ts/c (CD8) %	48.8 H%	17.7–41.1
Ts/c (CD8) no.	310/µl	292–1258
CD4:CD8	0.58 L	0.62–2.70
NK cells (CD16/56) %	15.7%	8.5–41.8
NK cells (CD16/56) no.	104 L/µl	158–1156

**Table 2.** Lymphocyte proliferation assay by [^3^H] thymidine

	Patient	Control	Reference range
Unstimulated cpm/10^6^ cells	5127 H	6500 H	<4303
PHA cpm/10^6^ cells	81 680 L	350 927	>189 770
Con A cpm/10^6^ cells	421 60 L	132 267	>86 170
PWM cpm/10^6^ cells	12 333 L	25 140	>24 258

Con A, concanavalin A; cpm, counts per minute; PHA, phytohemagglutinin; PWM, pokeweed mitogen.

## 3. Discussion

We present an interesting case of GS without severe hypogammaglobulinemia nor recurrent infections, contrary to typical manifestations of this syndrome, highlighting its heterogeneous presentation. Like our patient, most patients have undergone thymectomy before the diagnosis of GS [1, 6]. Development of thymoma is postulated to disrupt the balance between immunity and self-tolerance, leading to diverse clinical manifestations [3].

GS patients exhibit a diverse range of infection phenotypes, including defects in humoral, cellular, and phagocytic immunity. Although antibody deficiency is a hallmark of GS, predominant infections and mortality are related to cellular immunity defects [1, 3, 5]. These defects predispose patients to severe, recurrent infections by opportunistic organisms and reactivation of viruses such as CMV, which can manifest as pneumonia or colitis. Antibody deficiency contributes to recurrent sinopulmonary infections from encapsulated bacteria [3], with some patients later developing bronchiectasis [5, 6]. Chronic diarrhea is also common, associated with *Campylobacter* and *Salmonella*, CMV, and *Giardia* [3, 6]. Recent reports indicate that COVID-19 severity in GS patients varies from prolonged, mild infection and absent antibody response despite prior vaccination and infection, to severe, even fatal outcomes [7, 8].

Laboratory parameters in GS patients are equally heterogeneous. While hypogammaglobulinemia is a classical feature, many patients maintain relatively preserved Ig levels but functionally deficient Igs, as proven in our patient via pneumococcal vaccine challenges [6]. Lymphocyte enumeration commonly reveals low or absent B-cells, while T-cells and NK cells can remain variable [3]. Despite preserved T-cell counts, there is almost always in vitro evidence of cellular immune deficiency, illustrated by poor lymphocyte proliferation to mitogens and antigens [3, 5]. Our patient also showed a lack of response to protein-conjugated vaccines.

Autoimmunity is another prominent feature of GS, but autoimmune manifestations differ from those seen in B-cell deficient CVID patients [6]. In GS patients, myasthenia gravis with detectable antiacetylcholine receptor antibodies, pure red cell aplasia, and oral lichen planus are especially prevalent [3]. Many GS patients also develop neutralizing anti-cytokine autoantibodies to Type 1 interferons, IL-12, IL-17A/F, and IL-23, further weakening immune responses to various opportunistic and viral pathogens [9, 10]. The absence of recurrent infections in our patient may be attributed to the lack of these autoantibodies.

Because hypogammaglobulinemia, B-cell aplasia, and autoimmunity are not corrected following thymectomy, GS patients require continuous monitoring and management. Infections are managed with pathogen-specific antimicrobials, while long-term Ig replacement therapy effectively minimizes sinopulmonary infections. Routine vaccinations are recommended for prophylaxis. The development of autoimmunity should be closely monitored, especially affecting cell counts [5]. Our patient has remained infection-free for over a year while on subcutaneous Ig replacement therapy and CMV-prophylaxis with valganciclovir.

The prognosis of GS is generally poor. Aside from severe infections and autoimmune phenomena, gradual bone marrow failure is suggested, as evidenced by a progressive decline in lymphocytes, platelets, hemoglobin, and red blood cells over decades [5]. The combination of clinical features and laboratory findings is essential for the diagnosis of GS. Early recognition and appropriate management are crucial to prevent or manage the recurrent infections and complications associated with this rare immunodeficiency disorder. We therefore suggest that all patients with a history of thymoma may have Ig screened for the possibility of GS.

## Conflicts of interest

The authors have no financial conflicts of interest.

## Author contributions

Conceptualization: Valerie Chiang and Philip H. Li. Data curation: Valerie Chiang and Tiffany C. H. So. Investigation, visualization, and writing original draft: Valerie Chiang and Philip H. Li. Writing review and editing: Tiffany C. H. So and Philip H. Li. Final version: all authors approved.

## References

[1] Shi Y Wang C. When the Good syndrome goes bad: a systematic literature review. Front Immunol. 2021;12:679556. 34113351 10.3389/fimmu.2021.679556 PMC8185358

[2] Good RA Maclean LD Varco RL Zak SJ. Thymic tumor and acquired agammaglobulinemia: a clinical and experimental study of the immune response. Surgery. 1956;40:1010-1017. 13380673

[3] Sipos F Műzes G. Good’s syndrome: brief overview of an enigmatic immune deficiency. APMIS. 2023;131:698-704. 37729389 10.1111/apm.13351

[4] Bousfiha A Moundir A Tangye SG Picard C Jeddane L Al-Herz W Rundles CC Franco JL Holland SM Klein C Morio T Oksenhendler E Puel A Puck J Seppänen MRJ Somech R Su HC Sullivan KE Torgerson TR Meyts I. The 2022 update of IUIS phenotypical classification for human inborn errors of immunity. J Clin Immunol. 2022;42:1508-1520. 36198931 10.1007/s10875-022-01352-z

[5] Kabir A Polito V Tsoukas CM. Unraveling the natural history of Good’s syndrome: a progressive adult combined immunodeficiency. J Allergy Clin Immunol Pract. 2024;12:744-752.e3. 38122866 10.1016/j.jaip.2023.12.018

[6] Malphettes M Gérard L Galicier L Boutboul D Asli B Szalat R Perlat A Masseau A Schleinitz N Le Guenno G Viallard J-F Bonnotte B Thiercelin-Legrand M-F Sanhes L Borie R Georgin-Lavialle S Fieschi C Oksenhendler E; DEFicit Immunitaire de l’adulte Study Group. Good syndrome: an adult-onset immunodeficiency remarkable for its high incidence of invasive infections and autoimmune complications. Clin Infect Dis. 2015;61:e13-e19. 25828999 10.1093/cid/civ269

[7] Berzenji L Yogeswaran SK Snoeckx A Van Schil PE Wener R Hendriks JMH. Good’s syndrome and COVID-19: case report and literature review. Mediastinum. 2023;7:5. 36926289 10.21037/med-22-12 PMC10011861

[8] Lindahl H Smith CIE Bergman P. COVID-19 in a patient with Good’s syndrome and in 13 patients with common variable immunodeficiency. Clin Immunol Commun. 2021;1:20-24. 38620775 10.1016/j.clicom.2021.08.003 PMC8497938

[9] Burbelo PD Browne SK Sampaio EP Giaccone G Zaman R Kristosturyan E Rajan A Ding LI Ching KH Berman A Oliveira JB Hsu AP Klimavicz CM Iadarola MJ Holland SM. Anti-cytokine autoantibodies are associated with opportunistic infection in patients with thymic neoplasia. Blood. 2010;116:4848-4858. 20716769 10.1182/blood-2010-05-286161 PMC3321746

[10] Cheng A Kashyap A Salvator H Rosen LB Colby D Ardeshir-Larijani F Loehrer PJ Ding LI Lugo Reyes SO Riminton S Ballman M Rocco JM Marciano BE Freeman AF Browne SK Hsu AP Zelazny A Rajan A Sereti I Zerbe CS Lionakis MS Holland SM. Anti-interleukin-23 autoantibodies in adult-onset immunodeficiency. N Engl J Med. 2024;390:1105-1117. 38507753 10.1056/NEJMoa2210665 PMC12404335

